# Seeking coherence between ‘mobile learning’ applications and the everyday lives of medical residents

**DOI:** 10.1007/s40037-019-0519-0

**Published:** 2019-06-07

**Authors:** Diana Ramos, Roland Grad, Alenoush Saroyan, Peter Nugus

**Affiliations:** 1 Department of Family Medicine, McGill University, Montreal, Canada; 2 Department of Counseling and Educational Psychology, McGill University, Montreal, Canada; 3 Institute of Health Sciences Education and Department of Family Medicine, McGill University, Montreal, Canada

**Keywords:** Mobile learning technology, Educational technology, Medical residency, Spaced learning

## Abstract

**Introduction:**

The role of technology in health professions education has received increased research attention. Research has examined the interaction between humans and technology, focusing on the mutual influence between people and technology. Little attention has been given to the role of motivation and incentives in how learning technologies are used in relation to daily activities. This research aims to understand the relationship between medical-learning technology and its users.

**Methods:**

A mixed-method case study of a new medical-learning mobile application (app) for family medicine residents was undertaken at a Canadian university hospital. The Information Assessment Method is a custom-made app to help residents prepare for the College of Family Physicians of Canada licensing examination. Residents’ use of the app was tracked over a 7-month period and individual, semi-structured interviews were conducted with users. Data were thematically analyzed and correlated with app use data.

**Results:**

Factors identified as shaping residents’ mobile app use for learning, included: efficiency, mobility and resonance with life context; credibility of information retrieved; and relevance of content. Most influential was stage of residency. Second-year residents were more selective and strategic than first-year residents in their app use.

**Discussion:**

An emphasis on coherence between self-directed learning and externally dictated learning provides a framework for understanding the relationship between users and mobile-learning technology. This framework can guide the design, implementation and evaluation of learning interventions for healthcare professionals and learners.

## What this paper adds

There is little research conducted on mobile applications in health professions education (HPE), despite proliferation of their use in healthcare. There is even less guidance on how their use aligns with the everyday lives of the trainees who use them. This qualitative study shows that creative and strategic information use is a learning skill increasingly developed by medical trainees. ‘Coherence’, defined in terms of external influence (structure) and self-determination (agency), provides a framework for understanding and assessing technological innovations in HPE. Since second-year residents already make strategic use of such apps, special efforts must be made to inform first-year residents of how they can use such technology to support their learning. After all, they tend, relative to later-year residents, to be externally directed by learning technology, either using it rigidly, or abandoning it entirely.

## Introduction

A dramatic increase in the use of mobile phone applications (apps) among healthcare professionals has come about through evolving medical knowledge bases, the development and popularity of mobile communication devices, and the continually increasing specialization of medicine [1]. Currently, more than 7,000 health-related apps are available and used by healthcare professionals around the world [2]. Educational and social scientists have paid increased attention to the role of mobile technology in work and education [3]. Several studies have identified advantages of using mobile apps in clinical practice, including facilitation of communication, portability, and efficient time use [4]. In a recent study, most physicians reported using apps in their clinical practice [5]. Apps cover diverse topics and functions. The most commonly used are drug guides (79%), medical calculators (18%), coding and billing apps (4%), and calculator apps (e.g. pregnancy wheels) (4%). The most frequently downloaded apps are textbooks or reference materials (55%), classification and treatment algorithms (46%), and general medical knowledge compilations (43%) [6]. That clinicians are evidently downloading apps to assemble information to aid their practice suggests a burgeoning field of human-technology interaction for health professional education researchers to examine.

Social scientific research on how technology is used has generally focused on how humans interact with technology. The discipline of sociology, in particular, has drawn on its traditional distinction between ‘structure’ and ‘agency’ [3, 4, 7, 8]. This involves a focus on the extent to which human beings exercise freewill (agency), as opposed to being relatively bound by external (structural) constraints. More recently, social scientific studies have focused on the mutually influential relationship between humans and technology [3, 4, 7–10], where both human and non-human elements act and are acted upon. Our study draws on a framework of ‘sociomateriality’—a notion that assumes the mutual influence, or ‘entanglement’ of humans and technology [11, 12]. The idea is that a human being is part of a network in which they are influenced by what others do, in ways often unknowable to them [7–10]. The effects of this sociomaterial network on behaviour depend on the nature of the overlap between interests among individuals and their mutual relationships, how attracted particular individuals feel towards technology, and individuals’ shared cultural beliefs about technology [9–12]. Today, researchers tend to favour a view of ‘hybridity’ across humans and technology—that is, the co-identification of people with technologies and the ‘enmeshment’ of technologies with human bodies themselves [12, 13]. For instance, the response of a mobile-phone user to the ‘beep’ of a text message shows the potential hybridity between people and technology.

However, little research has focused on the relationship between mobile technology and users’ wider everyday contexts, and the extent to which apps match the situated actions that people perform in their everyday lives [13–15]. In general, the need for efficient mobility and time management are central, but under-explored, dimensions of technology use [13, 14]. Few studies have examined the role of motivation and incentives in the way learning technologies are used in relation to daily activities, including the relationship between intentional planning and spontaneous everyday action. Such research is needed if HPE research is to keep pace with innovations in learning technology. Moreover, few studies have examined the influence of mobile technology use on learning patterns in the context of formal exam preparation. This study aimed to understand the role of a learner’s everyday ‘mobile’ life in terms of the relationship between mobile medical learning technologies and users’ priorities.

## Methods

This paper focuses on the introduction of a new medical learning mobile application for family medicine residents at a Canadian university teaching hospital—the ‘Information Assessment Method (IAM)’ app—to optimize exam preparation. The IAM app was custom-made specifically based on the curriculum and licensing requirements of the College of Family Physicians of Canada (CFPC), to guide residents in preparation for the licensing examination. Sampled purposively, then, among other apps that are clinically based only, the app has links that specifically align with the definitions and details of topics and their corresponding objectives required for the CFPC physician licensing exam. Family medicine residents from a different institution chose the specific content. The content was reviewed and moderated by the second author, a family physician. The first author of the paper subsequently uploaded the content to the app.

The IAM app periodically alerts users to one of 99 clinical topics delineated by the College of Family Physicians of Canada as learning priorities for the final examination, which residents take to obtain a license. A weekly ‘prompt’ of one priority topic (also referred to as an ‘alert’ or ‘push notification’), reminds residents to study at regular intervals, in a ‘spaced’ fashion. The app’s design is based on ‘spaced learning theory,’ suggesting that distribution of learning spreads out study activities over time, improving the long-term acquisition of information [16]. We characterize the app’s ‘prompt’ to the user as an incursion by the external environment into the everyday, mobile life of the user, placing a demand on users to re-order their learning [16].

The residents’ responses to the app’s prompts were tracked throughout the study and their views elicited to understand the nature of its influence on their study patterns. This case study employed a mixed-methods approach and was conducted over a 7-month period (October–May 2015). Following approval by a university Institutional Review Board (otherwise known as a Human Research Ethics Committee), 20 family medicine residents (of a cohort of 46 invitees) consented to participate in the study. Eight participants were in their first year of residence, that is, postgraduate year 1 (PGY1) and 12 in their second year (PGY2). Inclusion criteria were limited to owning a smartphone with an iPhone (iOS 5.0 +) or Android (2.3 +) platform. Participants received instructions to download the IAM app onto their smartphones.

**Fig. 1:**
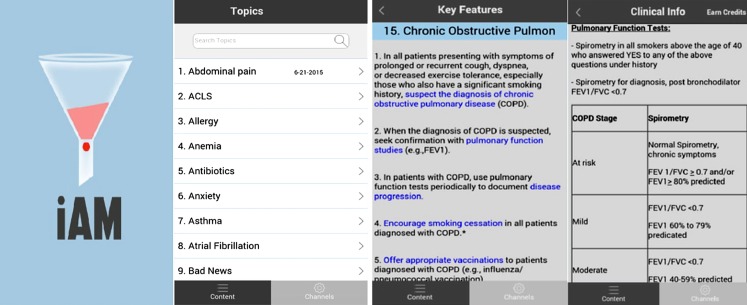
IAM app levels of information

Data sources included log files, which enabled us to track app use, and individual, open-ended, semi-structured interviews, which helped us to understand users’ experiences and perceptions of using an app to prepare for an exam that is an important milestone in their professional careers. To create the log files, a ‘hit’ was recorded whenever a user opened the app at the level of pages of clinical information (see Fig. 1). Every hit was logged as a separate entry in a password-protected database at www.99prioritytopics.ca. Log-file data included each participant’s name as well as the frequency, date, and time of page access.

The cut-off point of app use for inclusion in the study was 2 months (the length of two clinical rotations). This was because some clinical rotations have a greater workload than others, and 4 weeks (one clinical rotation) would not have been enough time to show the residents’ app use. All 20 participants installed the app. Based on the app use log file, participants were classified into three usage patterns: 3 were ‘non-users’ who installed the app but had no recorded hits; 9 were ‘discontinued users’, who had page hits but had stopped using the app ≥2 months before the study ended; and 8 were ‘persistent users,’ who had page hits and continued to use the app until the end of the study. Alerts were sent at the start of the weekly mandatory academic half-day, during which residents were released from clinical duties. The intention was to try and neutralize as a variable any possible patterns of difference in the busyness of the clinical work of either R1s or R2s.

A total of 15 semi-structured interviews were conducted with 13 participants representing each of the three levels of use. Two PGY2s were interviewed twice, based on the detail of their insights and the specificity of their feedback on the app’s technical functioning in the first interview. The interviews were evenly spaced over three stages of the data collection process to address the change in usage patterns of learning technology over time. Topics covered in the interviews included: perceptions of the app’s functionality, attitudes towards the app, perceptions of personal behaviours around the prompts, and perceptions of the relationship between the app and the participants’ learning preferences.

The interviews, featuring a combination of open questions (designed to elicit relatively unguided perceptions) and prompt questions (designed to seek more information about specific points raised) [17], were audio-recorded and transcribed verbatim. The software MaxQDA was used to organize the codes into which qualitative data were organized. The transcripts were analyzed independently by three coders who met to discuss and resolve a limited number (approximately 10%) of categorization differences. Thematic analysis was used to generate initial codes, compare and contrast data segments and organize segments into codes and then into categories, in order to conceptualize the relationships between the categories and develop conceptual themes [18]. Microsoft excel was used to calculate the descriptive statistics. The app use log files enabled triangulation with the data findings from interview responses.

## Findings

### The influence of learning level on app use

Several factors were identified as influential in determining the character of app use among participants. The most influential was *learning level*, measured across PGY1s and PGY2s. Clear differences were discernible in the degree of agency (i.e., freewill) residents demonstrated in using the app. Despite a baseline level of agency among all participants, app use by PGY1s could be characterized as ‘structured’ (i.e., guided by the app). PGY1s allowed the app itself to play a greater role in dictating how it would be used. When the app failed to align with their daily routine (i.e., when the prompt was unwelcome), they ceased using it entirely. A typical comment by PGY1s was:

*I [would like] to base my preparation on this app, cause it’s always nice to have one structure … just to follow [its] information. *(PGY1, Interview 1)

Thus, app use among PGY1s appeared to be relatively ‘externally/other-directed.’ Conversely, use by PGY2s could be characterized as more ‘agential’ or ‘self-directed.’ PGY2s took initiative in determining how they would use the app. Although there is evidence of sociomaterial influence of and on all app users, PGY2s appeared less troubled by the sometimes-unwelcomed interruptions of the prompt compared with PGY2s, simply ignoring the app on such occasions and opening it later without being prompted. They evinced more autonomy and strategy than the PGY1s in usage patterns:

*I would click on [the app] and I’ll read the topic of the week … for my own study purposes. [Only one] topic [per] week is nice now, but the closer I get to the exam, it’s [going to] be a topic [per] day. *(PGY2, Interview 9)

When asked whether they would follow the weekly prompts, one PGY2 said:

*Probably not. [I wouldn’t follow the prompts just because] an app is telling me that this week it’s ‘this topic’, which is arbitrary. [It] doesn’t make much sense to me.* (PGY2, Interview 7)

Log-file data seemed to match this concern, showing a higher rate of searching for topics other than the topic alerted for that week by PGY2s, compared with PGY1s. Tab. 1 illustrates the analysis of the tracked data.

**Table 1:** Usage duration by year of residency and number of participants

Year of residency	Usage frequency (based on number of participants)		Total number of participants
	2 ≤ months	3 ≥ months	
PGY 1	4	3	7
PGY 2	5	5	10
Total	9	8	17

*PGY* postgraduate year

**Fig. 2:**
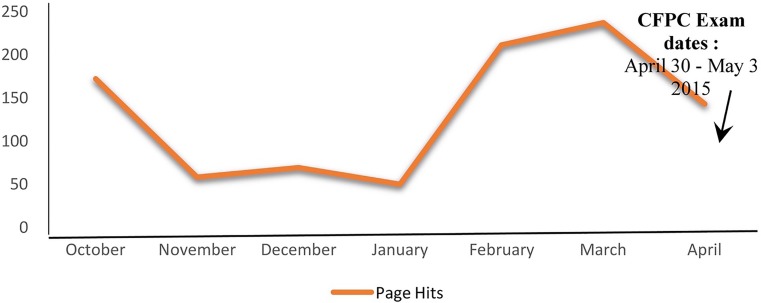
Monthly app use

These findings demonstrate the app’s perceived relevance and its strategic use by PGY2s. This signals a degree of command over the sociomaterial engagement between the app and its user. The app was designed to deliver regular prompts on a particular topic. However, the app’s use increased dramatically 3 months prior to the examination as illustrated in Fig. 2. This pattern points to a self-mandated use, especially by PGY2s, who might have been more motivated by a high-stakes professional examination.

### The influence of efficiency, mobility and resonance on app use

Underlying all factors affecting mobile app use in this study were *mobility* and *efficiency*. Users were more willing to initiate interactions with the app (i.e., respond to its prompting ‘beep’) if the app supported efficient use of their time, in building knowledge for the exam, and aligned with their physical mobility in accomplishing daily tasks. Framing use in general was the *resonance *of the app and its prompts with the routines and moment-to-moment priorities of each user. Reported app use was more likely to increase if its prompts aligned with the user’s daily life. Specifically, consistent app use was partially tied to residents’ need to efficiently prepare for a high-stakes exam within the 2 years of residency, while juggling competing personal and professional priorities. At a micro level, this also involved the need to be able to quickly manage (by reading or ignoring) the interruption the prompt posed in the flow of their daily lives:

*I mean, I don’t have a lot of time every night to do something, [but I aim to read] a little bit about it, like what the full history would be one night and exam and treatment, and kind of go through it like that.* (PGY1, Interview 6)

To be usable and used, then, the app needed to accommodate time-efficient use, complementing rather than impeding the user’s free mobility in their daily life. Free physical mobility was key to its use:

*[The app is] much more available [than other resources]. I have the phone with me all the time and it’s a good way to keep that there, at (my) fingertips. It’s great*. (PGY2, Interview 9)

Efficiency, which complements free physical mobility, was a criterion of use in the context of various other knowledge resources competing for the resident user’s attention:

*Usually, if I have time to prepare, I look at ‘UpToDate’ or ‘Essential Evidence,’ but as I get more used to [the IAM app], and if I find it helpful and it’s brief and to the point, then I would use that.* (PGY1, Interview 6)

Closely aligned to the need for efficient time use and user mobility, *ease of use* (i.e., confidence in using the technology) and *technological threshold* (i.e., tolerance for persevering in the face of the user’s technical uncertainty or the software’s technical faults) also aligned with the extent of app use. In general, the shared perception of ease of use and high technological threshold among the participants ensured that these variables did not strongly influence if, or how, residents used the app in this study. However, in some cases, technical hitches did impact app use. Technical hitches included the failure of the app to open in some iPhone operating systems, only opening reliably in Android form. The other hitch was that the app required internet use, and that internet coverage was often poor in hospitals. The impact, and potential impact, of technical faults underlines the importance of the app *resonating* with the users by aligning well with their daily routines:

*I was using [the app] at first. It was really, for me, the alarms and push notifications to guide me …. So when I stopped getting those push notifications … it wasn’t as useful to me.* (PGY2, Interview 13)

The failure of the app to open on some iPhone operating systems was fixed within a week of the app’s introduction. Indeed, only one person actually withdrew from the study on account of technical problems. In general, then, residents were apparently prepared to work hard to prepare for the exam and use the app to help them do so, but only when it met the functional needs of their everyday life, expressed in notion of mobility and reliability.

None of the participants reported problems to the research team prior to being asked specifically in the interviews. In the interviews, most participants reported minor technical hitches or questions. When asked how they sought to resolve technical issues, no one, including persistent users, reported approaching another resident to discuss it. The enmeshment between user and device appeared to be a solitary relationship, the residents reporting to work and solve problems in relative isolation from each other.

### The influence of credibility and relevance on app use

The *relevance* and *credibility* of the app, which were perceived to be high, reportedly increased use. Thus, these factors were persuasive in promoting mobile app use, while not strongly influential in determining the particular patterns of app use. The initial participant–app interaction evolved into an ongoing relationship for those users who perceived the app to be reliable and credible. Thus, reliability and credibility were factors in shaping the extent and nature of the sociomaterial entanglement of user and app. Although the sustainability of this trusting relationship was predicated, first and foremost, on relevance, users did not question the app’s relevance. The app was designed to help prepare residents for their certification examination, and as such their confidence in its relevance led to their participation in this study. Moreover, we found that establishing credibility relied, at least partly, on the users’ perceptions of the content’s trustworthiness and its currency. Both factors had an impact on app use:

*Will these topics be updated? [That’s important to me]. *(PGY1, Interview 1)

*If these could be updated regularly throughout the year, it would be in my best interest to go back and visit these more than once. *(PGY2, Interview 4)

When participants were asked what they thought about participating in shaping the content of the mobile app, contradictory comments emerged:

*Ideally, a resident, who has finished studying for his or her exam, could go through all the topics with their other resources … and see which topics were incomplete on the app.* (PGY2, Interview 15)

*I’m always scared with resident notes, because you’re always worried that something is … wrong.* (PGY2, Interview 11).

In terms of *learning approaches*, the following resident uniquely reflected on the app’s potential to facilitate collective learning among residents:

*[The app could] even encourage [us] to study in groups early on. [Because] that’s what I found to be the most helpful thing in preparing for the exam. … We could sit as a group with the app to make sure we hit all points.* (PGY2, Interview 11)

Thus, at least one participant considered the possibility that groups could delegate the organization of their collective learning to the app. However, this was the only resident who referred to group work or the use of the app with others, when asked how he or she would use the app to prepare for the exam. Despite the residents’ desire for group learning, for most participants the app seemed to support self-learning, thus, to some extent, exacerbating the tendencies of those already inclined to prepare, and deal with learning issues, alone:

*I think there were some people studying in groups. But I was more on my own. *(PGY2, Interview 12).

## Discussion

This paper has expanded previous literature featuring local experiments in design technology for medical trainees’ learning, and perceptions of usefulness. As a core assumption, the notion of sociomateriality accounts for the mutual and increasing entanglement of human beings with technology [11]. Very little was known about the way medical trainees actually use learning technology in their daily lives. This paper offers an account of the tension between structure (other-directed) and agency (user-directed) in navigating mobile-learning tools as a function of learning incentives and the developmental stages of learning. While it is increasingly less common to characterize human-technology interaction using the traditional sociological distinction between structure and agency [3, 4, 7, 8], its persistence is evident in the findings.

The app’s weekly prompt was intended to instil a habit of regular, structured learning—traditionally called ‘spaced learning’ or ‘spaced education’ [19, 20]—in its users, thus re-ordering their learning. Spaced learning contrasts with a mode of study colloquially known as ‘cramming’ or massed practice before an exam. In terms of the structure-agency distinction, PGY1s’ app use was externally governed by the learning mode and organization proposed by the app’s designer [21]. App use by PGY2s, on the other hand, was internally governed; and these users exhibited more agency and willingness to use the app if, when and how they desired. The observed differences in use patterns paralleled the difference in learning phases between PGY1s and PGY2s and the associated differences in their incentives for learning. Undoubtedly, PGY1s are more able to build knowledge cumulatively for its own sake, whereas PGY2s have the added pressure (or incentive) to pass a high-stakes physician-qualifying exam to be admitted as family medicine physicians.

The distinction between structure and agency is not fixed, but is mutual and recursive among both human and non-human elements [22]. It functions within a complex system of sociomaterial networks, in which a collection of elements deliver effects, rather than easily discernible causal relationships [8]. Accordingly, this study demonstrates both users’ influence on technology and technology’s influence on users, as part of an increasingly sociomaterial reality. This system of influence can be characterized as part of a search for *coherence* between individual will and external influences [23, 24].

As technology progressively permeates our lives, individuals try, within the constraints and opportunities of daily living and learning environments, to find a coherent, self-determined path [25, 26]. The success of the mutual entanglement between technology and its users depends on the extent to which the goals of technology designers and users intertwine. The study findings demonstrate the significance of the resonance of technology’s content and prompts with users’ priorities, technological thresholds, learning levels, perceptions of credibility and currency, and needs for efficient time use and mobility. Rather than relying strictly on the distinction between structure agency, accounting for structural elements through a lens of *coherence *can help us create a conceptual framework to evaluate the extent to which technology serves human needs. Such an assessment is vital in a field like healthcare, which aims to improve people’s lives through interventions, increasingly of a technological nature, and in which technology could easily become an end in itself.

We found that mobile learning applications may exacerbate learners’ isolation from one another. Medical students have been found to be particularly individualistic and competitive in the way they study and learn [27]. This is not necessarily undesirable. Yet, policy-makers and educators should consider incentives for learning, with a particularly critical eye on exam-driven behaviour and the culture that drives it. Such considerations are particularly salient given that the potential of mobile learning applications is apparently not being fully used to support collaborative learning among medical trainees [28]. Understanding the way learners interact with technology enables policymakers and educators to intervene, to foster learning beyond sociomaterial individualism, to learning through sociomaterial collaboration.

### Study limitations

A limitation of the study is the relatively small sample size and the collection of data among one specialty at one institution. Nevertheless, the study focused on the relatively generic subject of mobile learning among residents, suggesting broader resonance of its findings about how app use interacts with one’s personal life with residency trainees worldwide. After all, the topics discussed in interviews were not of a personal nature or idiosyncratic to the institution or region. Further research among other specialties and countries, and involving direct observation of mobile app use for learning, rather than reliance on second-hand reporting, would be illuminating.

## Conclusions

The framework of coherence emerging from this study can help clinician educators to shape exam preparation strategies, select mobile resources that resonate with students’ learning environments and needs, and guide the use and evaluation of mobile learning technologies according to learning levels. Such targeted interventions will promote agency within the mobile reality in which learners increasingly find themselves.

New clinical practice apps frequently appear on the market including, for example, calculators for risk stratification and treatment options. It is rare to find institutionally sanctioned apps devoted to preparation for licensing examinations. A rare exception is an app similar to the IAM app that was later developed by Western University in Ontario, Canada. Since this study, the IAM app has been integrated with the Western University’s app, which had a similar focus—training for the CFPC licensing examination. The IAM’s technical flaws have been remedied as part of this integration, and some of the content has been updated or otherwise revised. The app is not yet available for wider use. The efficacy of the combined apps awaits further study. Although residents sometimes study in groups, neither this nor other similar apps have acquired an inter-dependent online functionality—supporting sociomaterial collaboration—but this would be a valuable innovation.

Technology designers interested in mobile learning technology can use the factors identified in this study as influencing technology adoption to guide and evaluate the balance between end users’ needs with educators’ interests. We suggest that alerts from an app be provided to residents from their first year onwards. Further research into the culture of (and behaviours relating to) exam preparation can shed light on learning strategies and perceived learning gaps. Such research ought to provide guidance as to the most appropriate time during a training program to introduce particular educational interventions.
